# Potential for the development of *Taraxacum mongolicum* aqueous extract as a phytogenic feed additive for poultry

**DOI:** 10.3389/fimmu.2024.1354040

**Published:** 2024-03-11

**Authors:** Zhen Dong, Zhiqin Liu, Yufeng Xu, Bin Tan, Wenqing Sun, Qin Ai, Zihui Yang, Jianguo Zeng

**Affiliations:** ^1^ College of Veterinary Medicine, Hunan Agricultural University, Changsha, China; ^2^ Hunan Province Key Laboratory of Traditional Chinese Veterinary Medicine, Hunan Agricultural University, Changsha, China; ^3^ DHN Business Division, Wens Foodstuff Group Co., Ltd., Zhaoqing, China

**Keywords:** AGPs alternatives, antioxidant, broilers, growth performance, immune, intestinal flora, *Taraxacum mongolicum*

## Abstract

**Introduction:**

*Taraxacum mongolicum* (TM) is a kind of medicinal and edible homologous plant which is included in the catalogue of feed raw materials in China. It is rich in polyphenols, flavonoids, polysaccharides and other active substances, and shows many benefits to livestock, poultry and aquatic products. The study aimed to assess the potential of TM aqueous extract (TMAE) as a substitute for poultry AGPs.

**Methods:**

A total of 240 one-day-old Arbor Acker broilers were randomly assigned to four groups and fed a basal diet (Con) supplemented with 500, 1000, and 2000 mg/kg TMAE (Low, Medium, and High groups). The growth performance of the broilers was measured on day 21 and day 42. At the end of the trial, the researchers measured slaughter performance and collected serum, liver, spleen, ileum, and intestinal contents to investigate the effects of TMAE on serum biochemistry, antioxidant capacity, immune function, organ coefficient, intestinal morphology, flora composition, and short-chain fatty acids (SCFAs).

**Results:**

The results showed that broilers treated with TMAE had a significantly higher average daily gain from 22 to 42 days old compared to the Con group. Various doses of TMAE resulted in different levels of improvement in serum chemistry. High doses increased serum alkaline phosphatase and decreased creatinine. TMAE also increased the antioxidant capacity of serum, liver, and ileum in broilers. Additionally, middle and high doses of TMAE enhanced the innate immune function of the liver (IL-10) and ileum (Occludin) in broilers. Compared to the control group, the TMAE treatment group exhibited an increase in the ratio of villi length to villi crypt in the duodenum. TMAE increased the abundance of beneficial bacteria, such as *Alistipes* and Lactobacillus, while reducing the accumulation of harmful bacteria, such as *Colidextracter* and *Sellimonas*. The cecum's SCFAs content increased with a medium dose of TMAE. Supplementing broiler diets with TMAE at varying doses enhanced growth performance and overall health. The most significant benefits were observed at a dose of 1000 mg/kg, including improved serum biochemical parameters, intestinal morphology, antioxidant capacity of the liver and ileum, immune function of the liver and ileum, and increased SCFAs content. *Lactobacillus aviarius, norank_f_norank_o__Clostridia_UCG-014*, and *Flavonifractor* are potentially dominant members of the intestinal microflora.

**Conclusion:**

In conclusion, TMAE is a promising poultry feed additive and 1000 mg/kg is an effective reference dose.

## 1 Introduction

For over 60 years, antibiotic growth promoters (AGPs) have been used in livestock and poultry farming to prevent disease, promote growth, and reduce costs (1, 2). However, prolonged exposure to sub-therapeutic doses has significantly increased bacterial resistance to antibiotics, leading to pandemics in farmed animals, humans, and the environment. This poses significant obstacles and problems for livestock development and public health (3–5). In July 2020, China joined the United States, the European Union, and other regions in officially entering the era of feed prohibition (6). Commercial broilers often experience oxidative stress and intestinal flora disorder due to the imbalance between genetic improvement of carcass development and intestinal system development (7). Therefore, low-cost and effective alternatives to AGPs are crucial for the sustainable development of livestock production worldwide. Antibacterial peptides, probiotics, acidifiers, and plant active ingredients have been extensively researched and are considered promising alternatives to AGPs (8–11). However, the EU’s decades of experience in antibiotic-free farming demonstrate that AGPs cannot be replaced by a single product and require a more comprehensive strategy (12–14).

Plant-derived polyphenols and polysaccharides have been shown to provide various benefits to livestock and poultry, including immunomodulation, antioxidant, antibacterial, and anti-inflammatory effects (15–18). Certain phenolic compounds, such as flavonoids and functional polysaccharides, are not easily broken down and absorbed in the stomach and small intestine. Instead, they can reach the cecum intact and be fermented and utilized by the abundant intestinal microorganisms (19, 20). The phenolic compounds break down into smaller molecular weight phenols when absorbed by the body, providing a wide range of antioxidant effects. Additionally, during the decomposition process, they can affect the intestinal flora (21, 22). Some microbial species in the gut utilize functional polysaccharides as nutrients and ferment them to produce short-chain fatty acids, which positively impact the intestinal environment and help maintain its health (23, 24).

*Taraxacum mongolicum* (TM) is a perennial herb of the Asteraceae family that is distributed worldwide. It is commonly used in Chinese medicine to clear away heat and toxic materials, reduce swelling and disperse masses, and promote diuresis and treat stranguria. Recent pharmacological studies have demonstrated that it possesses antibacterial, antioxidant, antiviral, liver-protecting, and choleretic effects (25–28). It is commonly utilized for the prevention and treatment of respiratory and digestive tract ailments (29–31). TM is used as a vegetable and a substitute for tea in some countries due to its nutritional elements, such as vitamins, amino acids, minerals, and active secondary metabolites, such as polyphenols and sterols (32–34). In China, TM is also included in the list of feed ingredients. The study demonstrated that adding 500 mg/kg of dandelion powder to the broiler diet improved growth performance by enhancing the intestinal barrier and microbial composition (35). Additionally, Yang et al. (36) discovered that the water-soluble components of dandelion have a superior antioxidant effect and are more cost-effective and environmentally friendly as feed additives. However, the impact of TM aqueous extract (TMAE) on growth promotion in broilers and its possible mechanism of action have not been systematically evaluated. The International Symposium on Antibiotic Alternatives, held by the World Organization for Animal Health, emphasized the importance of considering the impact of the product on both the intestinal flora (biological barrier) and the intestinal barrier (physical and chemical barrier) (14). The hypothesis was that TMAE would enhance the antioxidant capacity, gut barrier function, gut microbiota composition, and short-chain fatty acids (SCFAs) in broilers, leading to improved growth performance. Thus, this study aimed to investigate the effects of dietary supplementation with TMAE on the growth performance, antioxidant capacity, intestinal barrier function, and intestinal microflora of broilers.

## 2 Materials and methods

### 2.1 Plant samples

The dried whole herb *Taraxacum mongolicum* Hand. - Mazz was purchased from Bozhou Medicinal Materials Market, Anhui Province and identified by Professor Yang Guangmin of Hunan University of Traditional Chinese Medicine. The samples are stored in Hunan Province Key Laboratory of Traditional Chinese Veterinary Medicine, Hunan Agricultural University.

### 2.2 Preparation of TMAE

The TMAE extract was prepared following the method described by Tan et al. (37). The extraction was carried out at 100 °C, three times for 50 minutes each, with a solid-liquid ratio of 1:8. The resulting filtrate was combined, concentrated, and dried to obtain the TMAE extract. The extract samples were analyzed for their contents of soluble sugars, flavonoids, and total phenols using chemical chromogenic methods, specifically the anthrone-sulfuric acid, alkaline nitrite-aluminum ions, and Folin-Ciocalteu methods. The commercial assay kits used in the analysis were provided by Beijing Boxbio Science & Technology Co., Ltd., China.

### 2.3 UHPLC-Q/TOF-MS analysis

Dissolve 4 mg of TMAE in 1 mL of 80% methanol (Merck) solution. After mixing, centrifuge the solution at 8000 r/min for 10 min and collect the supernatant. The supernatant samples were filtered using nylon membranes (13 mm × 0.22 μm, ANPEL Laboratory Technologies Inc.) and separated on an Agilent 1290 Infinity UHPLC (Agilent Technologies, Santa Clara, CA, USA) in series with an Agilent 6530 Accurate-Mass Q-TOF LC/MS (Agilent Technologies, Santa Clara, CA, USA) system equipped with Agilent Eclipse XDB-C18 (4.6 × 150 mm, 5 μm). The UHPLC-Q/TOF-MS test conditions are based on the method of Pieczykolan, A. et al. (38) with some modifications. The mobile phases used in this experiment were water containing 0.1% formic acid (solvent A) and acetonitrile containing 0.1% formic acid (solvent B). The gradient elution program followed the schedule below: 0-1.5 min, 13% B; 2-15 min, 20% B; 16-23 min, 25% B; 25-28 min, 33% B; 30-33 min, 60% B; 34-37 min, 13% B. The flow rate was 0.35 mL/min, and the column temperature was maintained at 25°C. The negative ion source voltage was set at -4.5 kV, the capillary temperature at 500°C, the spray gas at 55 psi, and the Curtain Gas at 30 psi. The mass-to-charge ratios of primary and secondary fragments of isolated compounds were screened in the MS-DIAL database and literature reports to identify compounds in extracts (39, 40).

### 2.4 Birds and experimental design

Prior to the experiment, the breeding ground and utensils were disinfected using formaldehyde fumigation. A total of 240 1-day-old Arbor Acres (AA) white feather broilers were randomly divided into four groups, with six replicates of ten broilers per replicate. The control group (Con) was fed a diet without TMAE, while the TMAE treatment groups were fed a basal diet supplemented with 500, 1000, and 2000 mg/kg TMAE (Low, Medium, and High). The experiment lasted for 42 days. The chickens were raised in 3-layer vertical cages, with 10 chickens in each cage. The temperature in the house was manually controlled at 37-32°C during the first week and decreased by 2°C per week from the second week. The relative humidity was maintained at 55%-70%, and the chickens had access to food and water at all times. Standardized daily management procedures for feeding and epidemic prevention were followed throughout the trial. The basic diet for white feather broilers is prepared using corn, soybean meal, soybean oil, and other raw materials in accordance with the nutritional requirements outlined in the Chicken Feeding Standard (NY/T 33-2004) (41). The feed is in powder form. **Table 1** shows the composition and nutritional levels of the basic diet. The study received approval from the Animal Ethics Committee of Hunan Agricultural University (Ethic approval number: CACAHU 20220922-1).

**Table 1:** Basic diet composition and nutrition level (air-dried basis).

Items	1 to 21 days of age	22 to 42 days of age
Raw material composition (%)		
Corn	55.23	61.00
Soybean meal	36.00	30.00
Soybean oil	4.6	5.20
L-lysine 55%	0.56	0.30
Methionine 98.5%	0.22	0.13
Calcium hydrogen phosphate	1.59	1.67
Limestone	1.20	1.10
Premix	0.60	0.60
Total	100.00	100.00
Nutrition level		
Metabolizable energy (MJ/kg)	12.76	13.18
Crude protein (%)	21.00	19.00
Lysine (%)	1.41	1.09
Methionine (%)	0.52	0.41
Cystine (%)	1.01	0.86
Calcium	0.90	0.84
Available phosphorus (%)	0.45	0.42

Premix provides per kg of complete diet: Copper sulfate pentahydrate: 70 mg, ferrous sulfate hydrate: 150 mg, zinc sulfate monohydrate: 300 mg, manganese sulfate monohydrate: 400 mg, Selenium: 50 mg, Iodine: 150 mg, Multivitamin: 300 mg, Choline: 500 mg, antioxidant: 100 mg, zeolite powder: 400 mg, fine bran: 380 mg, phytase: 200 mg, salt: 3 000 mg.

### 2.5 Sample collection

Body weight (BW), average daily gain (ADG), average daily feed intake (ADFI), and feed-to-gain ratio (F/G) were measured for each cage at 21 and 42 days of age. The chickens were fasted overnight but allowed to drink water. On day 42, the chickens were euthanized, and blood was collected using inert separating gel coagulation blood collection tubes. The blood was then centrifuged at 4 °C and 1500×g for 10 min, and the serum was collected and stored in centrifuge tubes. The thymus, spleen, and bursa of Fabricius were dissected, separated, and weighed after removing surface blood and water with absorbent paper. Tissue samples from the duodenum, jejunum, ileum, and cecum were collected and preserved in 4% paraformaldehyde fixative. The liver, duodenum, jejunum, and ileum samples were wrapped in tinfoil paper, frozen in liquid nitrogen, and stored in a -80 °C refrigerator. The contents of the duodenum, jejunum, ileum, and cecum were collected under aseptic conditions and stored in 2 mL cryotubes. The cryotubes were then frozen in liquid nitrogen and stored in a refrigerator at -80 °C. The slaughter performance was determined using the method specified in Poultry Production Performance Terminology and Measurement Statistical Methods (NY/T 823-2020) (42), and the calculation formula for each index is as follows: Carcass percentage, semi-eviscerated percentage, and eviscerated percentage are calculated as a percentage of broiler live weight. Carcass percentage is determined by dividing the carcass weight by the broiler live weight and multiplying by 100. Similarly, semi-eviscerated percentage is calculated by dividing the semi-eviscerated weight by the broiler live weight and multiplying by 100, while eviscerated percentage is calculated by dividing the eviscerated weight by the broiler live weight and multiplying by 100. The abdominal fat percentage (%) can be calculated by dividing the abdominal fat weight by the sum of the eviscerated weight and abdominal fat weight, and then multiplying the result by 100. Similarly, the breast muscle percentage (%) can be calculated by dividing the bilateral breast muscle weight by the eviscerated weight and multiplying the result by 100. Finally, the leg muscle percentage (%) can be calculated by dividing the bilateral leg muscle weight by the eviscerated weight and multiplying the result by 100.

### 2.6 Determination of serum biochemistry

An automatic serum biochemistry analyzer (ZY1280, Shanghai Kehua Bioengineering Co., Ltd., China) was used to determine the levels of aspartate aminotransferase (AST), lactate dehydrogenase (LDH), albumin (ALB), urea nitrogen (BUN), uric acid (UA), triglyceride (TG), low density lipoprotein (LDL-C), alanine aminotransferase (ALT), alkaline phosphatase (ALP), total protein (TP), globulin (GLB), glucose (GLU), creatinine (CRE), total cholesterol (TC), high-density lipoprotein (HDL-C), and creatine kinase (CK). The indicators were tested using commercial kits from Shanghai Kehua Bioengineering Co., Ltd., China.

### 2.7 Determination of antioxidant capacity

The antioxidant capacity of TMAE, serum, liver, and ileum were tested using a commercial assay kit from Shanghai Beyotime Biotechnology Co., Ltd., China and Abbkine Scientific Co., Ltd, China. The following parameters were determined using a full-wavelength multiplate reader (Infinite^®^ E Plex, Tecan Trading AG, Switzerland): total antioxidant capacity (T-AOC) based on ABTS and FRAP methods (43), total glutathione peroxidase (GSH-Px), superoxide dismutase (SOD), malondialdehyde (MDA), and catalase (CAT) (44).

### 2.8 Immune function assay

The thymus, spleen, and bursa of Fabricius were weighed, and the immune organ index was calculated using the formula: immune organ index % = 100 × immune organ weight/live body weight. Serum IgG, IgY, IgA, IgM, γ-IFN, and IL-10, as well as liver homogenate IgG and IL-10, were detected using a commercial solid-phase sandwich ELISA kit from Shanghai Enzyme-linked Biotechnology Co., Ltd., China. Serum IgG, IgY, IgA, IgM, γ-IFN, and IL-10, as well as liver homogenate IgG and IL-10, were detected using a commercial solid-phase sandwich ELISA kit from Shanghai Enzyme-linked Biotechnology Co., Ltd., China. Serum IgG, IgY, IgA, IgM, γ-IFN, and IL-10, as well as liver homogenate IgG and IL-10, were detected using a commercial solid-phase sandwich ELISA kit from Shanghai Enzyme-linked Biotechnology Co., Ltd., China. Additionally, lysozyme (LZM) and secretory IgA were measured. The ileum homogenate was analyzed for DEF β 1, Zonula Occluden-1 (ZO-1), Occludin, transferrin (TRF), Claudin-1, mucin 1 (MUC-1), mucin 2 (MUC-2), and transforming growth factor beta (TGF-β). To perform the analysis, 50 μL of either the standard or test sample was mixed with an equal volume of biotin-labeled antibody solution. The mixture was gently shaken and incubated at 37 °C for 45 minutes. Each reaction was thoroughly washed with wash solution, shaking for 30 seconds each time, and this process was repeated 4 times. To each well, add 100 μL of Horseradish Peroxidase-Streptavidin, shake well, and incubate at 37 °C for 30 minutes. After washing the reaction wells four times, add the substrate and let it react for five minutes in the dark. To terminate the reaction, add 50 μL of stop solution and measure the optical density (OD) value of each well at a wavelength of 450 nm.

### 2.9 Histological observation of intestinal tract

The tissues from the duodenum, jejunum, and ileum were fixed in formalin for 48 hours, rinsed with running tap water for 8 hours, dehydrated in a series of graded alcohol concentrations (70%, 80%, 90%, 95%, and 100%), transferred to xylene to make them transparent, and embedded in paraffin for pathology. The resulting paraffin blocks were cut into 6 μm sections using a microtome, stained with hematoxylin and eosin, and observed under a microscope. The study measured the length of the villi and the depth of the crypt using Olympus OlyVIA Image Viewer measurement tools (OLYMPUS Corporation, Tokyo, Japan). The length of the villi was defined as the vertical distance from the tip of the villi to the opening of the crypt, while the depth of the crypt was defined as the vertical distance from the opening of the crypt to its base.

### 2.10 Determination of SCFAs content

SCFAs in cecal contents were determined using the modified method of Zhang et al. (45). A 1 g sample of cecal content was weighed and mixed with 5 mL of ultrapure water for 30 min. The mixture was then centrifuged at 10000 rpm for 10 min after being left overnight at 4°C. The supernatant was transferred to a new container, and 4 mL of ultrapure water was added to the precipitate. The mixture was shaken and mixed for 30 min, then centrifuged again, and the supernatant was combined. The resulting supernatant was mixed with 25% metaphosphoric acid (v:v=9:1) to determine SCFAs. The sample was passed through a 45 μm microporous membrane and then analyzed for SCFAs, including acetic acid (aa), propionic acid (pa), isobutyric acid (iba), butyric acid (ba), isovaleric acid (iva), and valeric acid (va), using a SHIMADZU GC-2010plus gas chromatograph (SHIMADZU Corporation, Kyoto, Japan) equipped with a DB-FFAP column (0. 25 μm × 30 m × 250 μm) (Agilent Technologies Inc., Santa Clara, USA). Chromatographic conditions were as follows: The sample was heated to 70°C for 3 minutes and then programmed to reach 210°C at a rate of 5°C/min and held for 10 minutes. The vaporization chamber temperature was set to 230°C and the FID detector temperature was set to 280°C. High purity nitrogen (purity ≥ 99.999%) was used as the carrier gas with a flow rate of 1 mL/min. The injection volume was 2.0 μL with a split ratio of 50:1.

### 2.11 16S rRNA sequencing of intestinal microbes

The small intestine samples were prepared by mixing equal amounts of duodenum, jejunum, and ileum contents. The quality of the extracted DNA was tested using 1% agarose gel electrophoresis and the DNA concentration and purity were determined using a BioPhotometer D30 (Eppendorf, Hamburg, Germany), following the TIANamp Stool DNA Kit (TIANGEN BIOTECH (BEIJING) CO., LTD.) protocol from Beijing, China. The 16S rDNA’s V4-V16 region was amplified through PCR using 338F (5’-ACTCCTACGGGAGGCAGCAG-3’) and 806R (5’-GGACTACHVGGGTWTCTAAT-3’). The PCR products of each sample were mixed and recovered through 2% agarose gel. The products were then purified using the TIANgel Purification Kit and TIANquick Midi Purification Kit (TIANGEN BIOTECH (BEIJING) CO., LTD.), Beijing, China. The purified products were detected through 2% agarose gel electrophoresis and quantified using the Quantus™ Fluorometer (Promega, USA). The TIANSeq DirectFast Library Kit (TIANGEN BIOTECH (BEIJING) CO., LTD., Beijing, China) was used to prepare the samples. Sequencing was performed by Shanghai Majorbio Biopharm Technology Co., Ltd. (Shanghai, China) using Illumina’s MiseqPE300 platform.

Fastp (v 0.19.6) was used for quality control of raw sequencing sequences, while splicing was performed using Flash (v 1.2.11). Sequences were clustered into OTUs using Uparse (v 11) with a similarity threshold of 97%, and mosaics were rejected. The OTU representative sequences were annotated using the RDP Classifier (v 2.13) based on the silva138/16s_bacteria taxonomic database, with a confidence threshold of 0.7 for taxonomic annotation results. The results were analyzed using Majorbio Cloud Platform (https://cloud.majorbio.com). The analysis included dilution curve analysis, alpha diversity analysis (Shannon index and Simpson index), species composition analysis (community composition analysis Bar plot), beta diversity analysis (principal co-ordinates analysis, PCoA), and species difference analysis (Kruskal-Wallis test, Kruskal-Wallis H test, and LDA discriminant results table).

### 2.12 Data analysis

Duncan’s multiple comparison test was used to analyze the results of growth performance, slaughter performance, serum biochemistry, antioxidant capacity, immune function, intestinal morphology, and SCFAs using IBM SPSS Statistics 26.0 (IBM Corporation, Armonk, USA). A significance level of P<0.05 was considered statistically significant. The data is presented as mean ± pooled SEM. The analytical plots for gut microbiota were generated using the R package. GraphPad Prism 8.0.2 (GraphPad Software, San Diego, USA) was used to generate line graphs displaying TMAE *in vitro* antioxidant results.

## 3 Results

### 3.1 Active ingredient content and composition of TMAE

The chemical chromogenic method was used to determine the content of active substances in TMAE. The TMAE extract had a yield of 39.28%. The total phenols content was 0.64%, the flavonoids content was 3.22%, and the soluble sugar content was 19.03%.

Fourteen phenolic compounds were identified from TMAE by UPLC-Q-TOF-MS, including chicory acid, caffeoyl tartaric acid, caffeic acid, luteolin, and its derivatives. The main phenolic compounds of TMAE were 1,3-dihydroxyacetone dimer, L-malic acid, luteolin 7-rutinoside, and chicoric acid, as shown in **Figure S1** and **Table S1**.

### 3.2 Growth performance

**Table 2** shows the results of the effect of adding TMAE to the diet on the growth performance of broilers. At 21 days of age, the BW of broilers in the Con group was significantly higher than that in the TMAE groups (P<0.05). However, at 42 days of age, the BW of broilers in the TMAE groups was significantly higher than that in the Con group (P<0.05). The low-dose group showed the most significant weight gain. From 1 to 21 days of age, the average daily gain (ADG) of broilers in the Con group was significantly higher than that in the TMAE group (P<0.05). Conversely, from 22 to 42 days of age, the ADG of broilers in the TMAE group was significantly higher than that in the Con group (P<0.05).

**Table 2:** Effects of dandelion aqueous extract on production performance of broilers (n=12).

Item	Group				SEM	P value		
	Con	Low	Medium	High		ANOVA	Linear	Quadratic
Body weight at 1 day of age (g)	41.02	41.04	40.99	41.03	0.01	0.364	0.727	0.737
Body weight at 21 days of age (g)	578.70 ^a^	562.96 ^b^	542.69 ^c^	547.74 ^c^	2.15	0.000	0.000	0.008
Body weight at 42 days of age (g)	1917.72 ^c^	2149.00 ^a^	2074.00 ^b^	2028.00 ^b^	14.03	0.000	0.029	0.000
1 to 21 days of age								
Average daily feed intake (g/d)	32.61	35.04	34.53	34.81	0.47	0.217	0.144	0.252
Average daily gain (g/d)	25.60 ^a^	24.85 ^b^	23.89 ^c^	24.13 ^c^	0.11	0.000	0.000	0.011
Feed-to-gain ratio	1.28	1.41	1.45	1.44	0.02	0.101	0.040	0.194
22 to 42 days of age								
Average daily feed intake (g/d)	107.44	121.38	121.62	116.53	2.97	0.272	0.294	0.118
Average daily gain (g/d)	63.76 ^c^	75.53 ^a^	72.92 ^ab^	70.49 ^b^	0.65	0.000	0.001	0.000
Feed-to-gain ratio	1.69	1.60	1.68	1.65	0.03	0.756	0.838	0.678
1 to 42 days of age								
Average daily feed intake (g/d)	70.02	78.21	78.07	75.67	1.52	0.161	0.198	0.081
Average daily gain (g/d)	44.27 ^c^	50.19 ^a^	48.40 ^ab^	47.31 ^b^	0.35	0.000	0.009	0.000
Feed-to-gain ratio	1.36	1.21	1.26	1.23	0.02	0.154	0.135	0.230

Data in the same row with no or same superscript letters indicate non-significant differences (P > 0.05), and different letters indicate significant differences (P < 0.05).

### 3.3 Slaughter performance

**Table 3** shows the results of the effect of TMAE on the slaughter performance of broilers. The slaughter performance of the TMAE groups was not significantly different from that of the Con group.

**Table 3:** Effect of TMAE on slaughter performance of broilers (n=12).

Item (%)	Group				SEM	P value		
	Con	Low	Medium	High		ANOVA	Linear	Quadratic
Carcass percentage	93.81	92.84	91.57	92.42	0.46	0.427	0.213	0.336
Semi-eviscerated percentage	85.22	84.76	86.35	85.10	0.50	0.732	0.550	0.309
Eviscerated percentage	72.80	74.84	74.24	72.41	0.38	0.063	0.032	0.651
Breast muscle	20.86	22.77	22.31	19.93	0.45	0.085	0.053	0.902
Leg muscle percentage	14.20	14.48	15.74	15.01	0.27	0.191	0.287	0.210
Abdominal fat percentage	0.60	0.53	0.85	1.30	0.12	0.059	0.029	0.244

Data in the same row with no or same superscript letters indicate non-significant differences (P > 0.05), and different letters indicate significant differences (P < 0.05).

### 3.4 Serum biochemistry

The results of the effect of TMAE supplementation in the diet on serum biochemistry of broilers are shown in **Table 4**. The results showed that ALP in High group was significantly higher than that in Con group (P<0.05). BUN was significantly increased in Medium group (P<0.05). TG was significantly lower in Low group (P<0.05). HDL in Medium group was significantly higher than that in control group (P<0.05). The serum CRE in High group was significantly lower than that in control group (P<0.05). There was no significant effect on other indexes.

**Table 4:** Effect of TMAE on serum biochemistry of broilers (n=12).

Item	Group				SEM	P value		
	Con	Low	Medium	High		ANOVA	Linear	Quadratic
AST (U/L)	405.41	427.32	479.92	477.42	21.50	0.505	0.163	0.786
ALT (U/L)	8.25	8.97	10.87	11.46	0.60	0.157	0.027	0.959
LDH (U/L)	2642.07	2490.63	2985.67	3015.00	88.95	0.154	0.056	0.627
ALP (U/L)	1297.04 ^b^	1078.89 ^b^	1040.43 ^b^	2299.41 ^a^	104.74	0.000	0.000	0.000
BUN (mmol/L)	0.30 ^bc^	0.27 ^c^	0.44 ^a^	0.40 ^ab^	0.02	0.020	0.008	0.857
UA (μmol/L)	173.86	217.71	236.40	195.80	9.94	0.107	0.341	0.049
TG (mmol/L)	1.56 ^a^	0.52 ^b^	1.18 ^a^	1.48 ^a^	0.11	0.001	0.571	0.000
LDL (mmol/L)	0.89	1.01	1.16	1.12	0.05	0.235	0.065	0.431
HDL (mmol/L)	2.18 ^b^	1.98 ^b^	3.18 ^a^	2.47 ^b^	0.11	0.002	0.016	0.212
TP (g/L)	36.18	44.46	39.58	38.80	1.61	0.386	0.83	0.189
GLB (g/L)	24.14	29.55	28.06	26.09	1.06	0.286	0.628	0.103
ALB (g/L)	12.12	14.78	14.44	12.79	0.54	0.234	0.723	0.06
GLU (mmol/L)	12.12	11.09	12.34	10.99	0.44	0.267	0.164	0.920
CRE (μmol/L)	2217.46 ^a^	1863.83 ^a^	1817.46 ^a^	1125.10 ^b^	107.35	0.000	0.000	0.327
TC (mmol/L)	3.14	3.02	3.47	3.48	0.12	0.475	0.156	0.801

Data in the same row with no or same superscript letters indicate non-significant differences (P > 0.05), and different letters indicate significant differences (P < 0.05).

### 3.5 Antioxidant capacity

**Figure 1:**
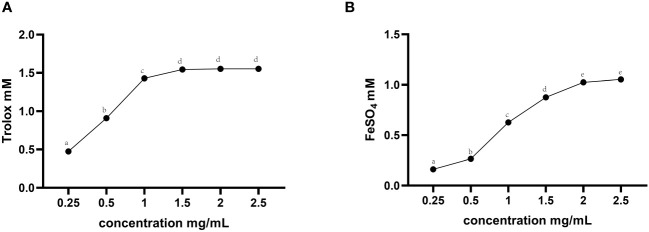
Total antioxidant capacity results for TMAE. **(A)** ABTS free radical scavenging ability; **(B)** Iron ion reduction antioxidant capacity. Data in the same row with no or same superscript letters indicate non-significant differences (P > 0.05), and different letters indicate significant differences (P < 0.05).

The *in vitro* total antioxidant capacity of TMAE was determined by measuring its ABTS free radical scavenging capacity and iron ion reducing antioxidant capacity. The study found that the free clearance capacity of ABTS reached its maximum at a concentration of 1.5 mg/mL of TMAE, resulting in a Trolox-Equivalent Antioxidant Capacity (TEAC) of 1.54 mM Trolox/1.5 mg/mL (**Figure 1A**). Additionally, the antioxidant capacity of TMAE increased with concentration in the tested range for the FRAP test, but the upward trend stabilized after reaching 2 mg/kg. The FRAP assay revealed a maximum antioxidant capacity of approximately 1.02 mM FeSO4/2 mg/mL (**Figure 1B**). The TMAE groups exhibited significantly higher serum total antioxidant capacity and CAT activity compared to the Con group (P<0.05). Additionally, the liver in the Low group showed significantly higher ABTS free radical scavenging ability than the control group (P<0.05). The liver’s total antioxidant capacity (FRAP) significantly increased (P<0.05) in the TMAE treatment group, while the content of MDA significantly decreased (P<0.05). The activity of CAT in the liver of broilers in the Medium group also significantly increased (P<0.05). Additionally, GSH-Px activities in the liver and ileum of broilers in the High group significantly increased (P<0.05) (**Table 5**).

**Table 5:** Effect of TMAE on antioxidant ability of broilers (n=12).

Item	Group				SEM	P value		
	Con	Low	Medium	High		ANOVA	Linear	Quadratic
Serum								
ABTS (mM)	1.13	0.97	1.04	1.01	0.03	0.255	0.261	0.241
FRAP (mM)	1.51 ^b^	2.01 ^a^	1.88 ^a^	2.05 ^a^	0.06	0.000	0.015	0.019
MDA (μM)	0.93	0.88	0.78	0.86	0.05	0.839	0.529	0.621
CAT (nmol/min/mL)	10.80 ^b^	23.28 ^a^	30.49 ^a^	29.47 ^a^	2.65	0.015	0.004	0.115
GSH-Px (U/mL)	644.60	648.29	641.19	642.48	1.41	0.302	0.292	0.669
SOD (units)	1.40	1.46	1.35	1.39	0.04	0.853	0.763	0.889
Liver								
ABTS (mmol/g)	0.23 ^b^	0.27 ^a^	0.24 ^b^	0.25 ^ab^	0.01	0.015	0.548	0.058
FRAP (mmol/g)	0.15 ^b^	0.18 ^a^	0.19 ^a^	0.18 ^a^	0.00	0.001	0.002	0.002
MDA (µmol/mg)	200.70 ^a^	117.41 ^b^	125.55 ^b^	124.23 ^b^	9.91	0.007	0.042	0.019
CAT (nmol/min/mL)	27.46 ^b^	33.63 ^b^	49.57 ^a^	34.46 ^b^	2.40	0.002	0.003	0.008
GSH-Px (U/mL)	91.16 ^b^	136.62 ^b^	151.22 ^ab^	180.82 ^a^	8.32	0.001	0.620	0.544
SOD (U/mg)	175.30	267.14	202.48	220.24	15.90	0.215	0.134	0.240
Ileum								
ABTS (mmol/g)	0.52	0.54	0.55	0.52	0.01	0.569	0.901	0.177
FRAP (mmol/g)	2.48	2.48	2.37	2.45	0.03	0.502	0.407	0.263
MDA (µmol/mg)	11.55	9.79	10.33	11.81	0.37	0.182	0.701	0.035
CAT (nmol/min/mL)	17.47	17.84	17.40	14.84	0.54	0.152	0.091	0.166
GSH-Px (U/mL)	206.69 ^c^	262.55 ^bc^	283.14 ^ab^	333.09 ^a^	13.29	0.004	0.802	0.519
SOD (U/mg)	112.07	111.59	110.20	117.86	5.58	0.966	0.756	0.737

Data in the same row with no or same superscript letters indicate non-significant differences (P > 0.05), and different letters indicate significant differences (P < 0.05).

### 3.6 Immune function

The study results suggest that dietary supplementation with TMAE did not significantly affect immune organ indices in broilers (refer to **Table 6**). Furthermore, the ELISA test results of serum, liver, and ileum tissue samples (refer to **Table 7**) showed that TMAE treatment did not significantly affect serum immune indexes of broilers. However, the medium and high doses of TMAE significantly increased IL-10 content in the liver and Occludin content in the ileum (P<0.05). The group with higher levels also showed a significant increase in the expression of LZM, sIgA, TRF, ZO-1, and DEF β1 in the ileum (P<0.05).

**Table 6:** Effect of TMAE on immune organ index of broilers (n=12).

Item %	Group				SEM	P value		
	Con	Low	Medium	High		ANOVA	Linear	Quadratic
Thymus index	0.20	0.19	0.21	0.23	0.01	0.633	0.224	0.651
Spleen index	0.13	0.13	0.11	0.16	0.01	0.129	0.220	0.112
Index of bursa of Fabricius	0.26	0.23	0.17	0.20	0.02	0.242	0.126	0.342

Data in the same row with no or same superscript letters indicate non-significant differences (P > 0.05), and different letters indicate significant differences (P < 0.05).

**Table 7:** Effect of TMAE on immune function of serum, liver and ileum of broilers.

Item (μg/mL)	Group				SEM	P value		
	Con	Low	Medium	High		ANOVA	Linear	Quadratic
Serum								
IL-10	0.06	0.07	0.06	0.06	0.00	0.121	0.397	0.057
IGA	45.28	47.65	46.50	49.46	0.60	0.082	0.032	0.800
IGY	55.10	56.52	56.41	56.57	1.09	0.962	0.676	0.785
IFN-γ	11.58	11.77	11.85	11.63	0.21	0.970	0.909	0.645
IGG	0.06	0.06	0.06	0.06	0.00	0.335	0.072	0.862
Liver								
IL-10	0.67 ^b^	0.69 ^ab^	0.69 ^a^	0.70 ^a^	0.00	0.022	0.004	0.283
IGG	0.61	0.61	0.61	0.61	0.00	0.375	0.750	0.566
Ileum								
Claudin-1	5.26	5.35	5.31	5.91	0.10	0.087	0.037	0.205
LZM	59.85 ^b^	62.81 ^b^	61.43 ^b^	72.35 ^a^	1.12	0.000	0.000	0.011
Occludin	59.18 ^b^	59.16 ^b^	65.60 ^a^	68.40 ^a^	1.23	0.007	0.001	0.505
sIgA	58.15 ^b^	59.03 ^b^	60.56 ^b^	68.81 ^a^	0.96	0.000	0.000	0.005
TRF	62.14 ^b^	59.08 ^b^	60.50 ^b^	74.04 ^a^	1.27	0.000	0.000	0.225
ZO-1	62.73 ^b^	65.35 ^ab^	65.74 ^ab^	71.41 ^a^	1.14	0.042	0.008	0.469
DEF β1	53.62 ^c^	54.72 ^b^	51.19 ^bc^	58.99 ^a^	0.75	0.000	0.001	0.006
MUC-1	7.49	7.70	7.70	7.64	0.14	0.625	0.943	0.625
MUC-2	3.61	3.47	3.42	3.73	0.06	0.102	0.266	0.069
TGF-β	38.45 ^b^	42.85 ^a^	39.77 ^ab^	43.84 ^a^	1.21	0.067	0.052	0.020

Data in the same row with no or same superscript letters indicate non-significant differences (P > 0.05), and different letters indicate significant differences (P < 0.05).

### 3.7 Histological morphology of small intestine

**Table 8** shows the effect of TMAE supplementation on small bowel morphology. Compared to the Con group, the Low, Medium, and High groups had significantly increased villus length and villus-to-crypt ratio in the duodenum (P<0.05). Additionally, the Low group had significantly increased villus length and villus-to-crypt ratio in the ileum (P<0.05).

**Table 8:** Effects of TMAE on intestinal morphology of broilers (n=12).

Item (μm)	Group				SEM	P value		
	Con	Low	Medium	High		ANOVA	Linear	Quadratic
Duodenal								
Villus length	1162.28 ^b^	1768.04 ^a^	1619.72 ^a^	1632.76 ^a^	54.42	0.000	0.000	0.028
Crypt depth	167.55	190.04	175.63	153.42	5.37	0.085	0.053	0.174
Villus to crypt ratio	6.36 ^b^	9.33 ^a^	9.12 ^a^	10.18 ^a^	0.41	0.007	0.021	0.016
Jejunum								
Villus length	1198.11	1274.69	1120.04	1170.63	30.40	0.41	0.53	0.18
Crypt depth	159.17	140.11	152.15	131.50	4.31	0.08	0.33	0.35
Villus to crypt ratio	7.73	9.18	8.21	9.14	0.29	0.25	0.21	0.72
Ileum								
Villus length	776.02 ^b^	999.69 ^a^	853.01 ^b^	827.78 ^b^	27.07	0.030	0.006	0.345
Crypt depth	122.23	161.12	124.21	126.99	6.50	0.108	0.053	0.205
Villus to crypt ratio	6.16 ^b^	9.17 ^a^	7.79 ^ab^	6.73 ^b^	0.38	0.045	0.007	0.573

Data in the same row with no or same superscript letters indicate non-significant differences (P > 0.05), and different letters indicate significant differences (P < 0.05).

### 3.8 SCFAs

**Table 9** displays the SCFAs content in the cecal contents of broilers. The content of aa and ba in the cecal contents was significantly increased in the Medium group (1000 mg/kg) with the addition of TMAE compared to the Con group (P<0.05).

**Table 9:** Effects of TMAE on SCFAs in cecum of broilers (n=12).

Item (μg/g)	Group				SEM	P value		
	Con	Low	Medium	High		ANOVA	Linear	Quadratic
Acetic acid	4713.96 ^b^	4412.68 ^b^	5805.06 ^a^	3729.72 ^b^	217.72	0.002	0.314	0.014
Propionic acid	1208.27	1083.95	985.98	971.27	41.89	0.166	0.047	0.494
Isobutyric acid	168.99	210.04	185.53	156.88	9.95	0.249	0.494	0.088
Butyric acid	740.90 ^bc^	921.57 ^ab^	990.65 ^a^	594.73 ^c^	49.00	0.004	0.281	0.001
Isovaleric acid	205.84	209.86	211.64	163.79	10.61	0.311	0.217	0.232
Valeric acid	135.53	140.70	156.60	116.78	7.67	0.324	0.569	0.155

Data in the same row with no or same superscript letters indicate non-significant differences (P > 0.05), and different letters indicate significant differences (P < 0.05).

### 3.9 Gut microbiota composition

#### 3.9.1 Microbiological analysis of small intestine

**Figure 2:**
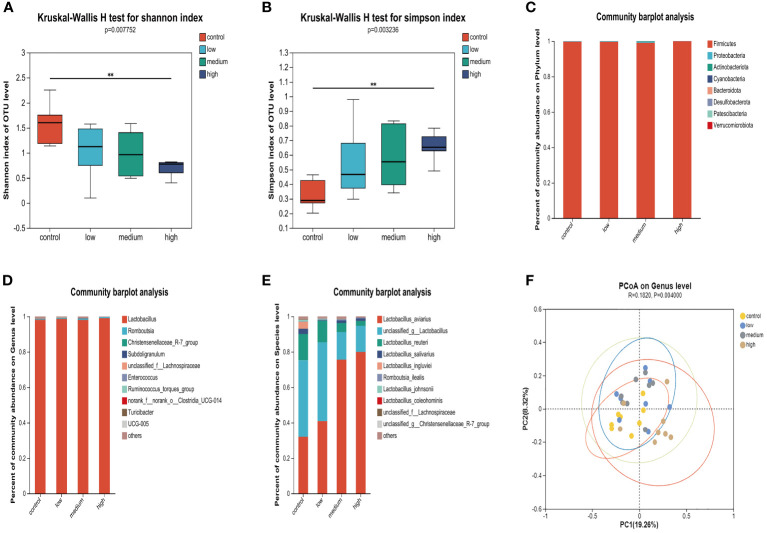
Effect of TMAE supplementation on small intestinal microflora of broilers. **(A, B)** Effect of TMAE on intestinal microbial alpha diversity (Shannon and Simpson index); **(C–E)** Colony composition of small intestinal microorganisms at phylum, genus and species levels after TMAE addition; **(F)** Intestinal microbial beta diversity analysis.

The results of the α diversity analysis (**Figures 2A, B**) indicate that at the OUT level, the Shannon index of microorganisms in the small intestine of the Con group was significantly higher than that of the TMAE treatment groups (P<0.05). Additionally, the Simpson index was significantly lower in the Con group compared to the TMAE treatment group (P<0.05). To analyze the effect of dietary TMAE supplementation on microbial community composition, we analyzed the microbial community of each dose group at the phylum, genus, and species levels. *Firmicutes* was the dominant phylum in all small intestine samples, accounting for over 99% (**Figure 2C**). At the genus level, the intestinal flora was relatively simple, with *Lactobacillus* being the main genus, accounting for over 98% (**Figure 2D**). At the species level, it was observed that *Lactobacillus aviarius* was gradually enriched in the small intestine with the addition of TMAE (**Figure 2E**). Cluster analysis of the population was performed using PCoA (**Figure 2F**), which showed that dietary TMAE supplementation did not have a significant effect on the microbial composition of the small intestine at the genus level or higher.

#### 3.9.2 Microbiological analysis of cecum

**Figure 3:**
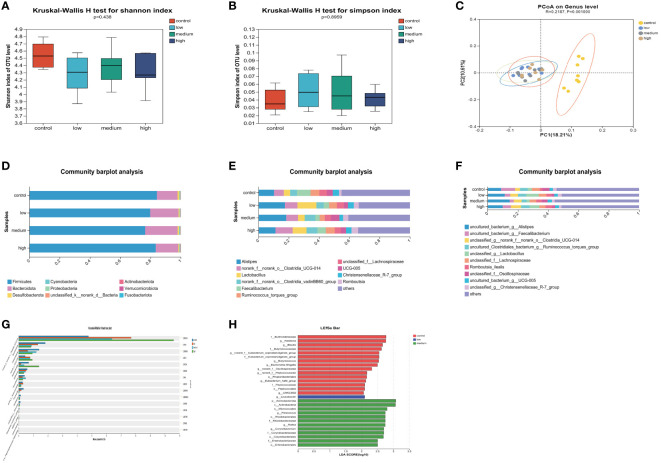
Effect of TMAE supplementation on cecal microorganism of broilers. **(A, B)** Effect of TMAE on Cecal Microbial Alpha Diversity (Shannon and Simpson index); **(C)** Cecal microbial beta diversity analysis; **(D–F)** Colony composition of cecal microorganisms at phylum, genus and species levels after TMAE addition; **(G)** Differentiation of enterobacteria at the genus level using the Kruskal-Wallis H test; **(H)** Effect of TMAE on Linear Discriminant Analysis Effect Size (LEfSe).

The α diversity of the cecal microbiota was not significantly affected (P>0.05) by the addition of TMAE to the diet (**Figures 3A, B**). The dominant flora in the cecum consisted mainly of *Firmicutes* and *Bacteroidetes*, which accounted for more than 97% of the total. The proportion of Bacteroidetes increased in the low and medium dose groups of TMAE compared to the Con group (**Figure 3D**). The cecal flora is primarily composed of *Alistipes*, *norank_f:norank_o:Clostridia_UCG-014*, *Lactobacillus*, *norank_o:Clostridia_vadin BB60_group*, *Faecalibacterium*, *Ruminococcus_torques_group*, *unclassified_f_Lachnospiraceae*, *UCG-005*, *Christensenellaceae_R-7_group*, and *Romboutsia* (**Figure 3E**). The species-level composition is generally consistent with that of the genus level (**Figure 3F**). PCoA analysis revealed that the cecal microbiota of the Con and TMAE treated groups formed two distinct clusters (**Figure 3C**), indicating significant differences in microbial communities. This suggests that dietary supplementation with TMAE had varying effects on the cecal microflora. The Kruskal-Wallis H test (**Figure 3G**) was used to explore the flora that differed significantly between the different groups at the genus level. The relative abundance of *norank_f:norank_o:Clostridia_UCG-014* and *norank_f:UCG-010* was found to be significantly increased (P<0.05) using the Kruskal-Wallis H test and LEfSe Multi-level Species Difference Discriminant Analysis (LDA Linear Discriminant). The abundance of *Butyricicoccus*, *Colidextrebacter*, *Sellimonas*, and *Fournierella* was significantly decreased (P<0.05) in the TMAE-treated group compared to the control group. Parabacteroides showed significant decreases and increases in the low and medium dose groups (P<0.01), but not in the high dose group (**Figure 3H**). *Flavonifractor* increased significantly in the medium dose group (P<0.05).

#### 3.9.3 Correlation analysis between cecal microorganisms and SCFAs

**Figure 4:**
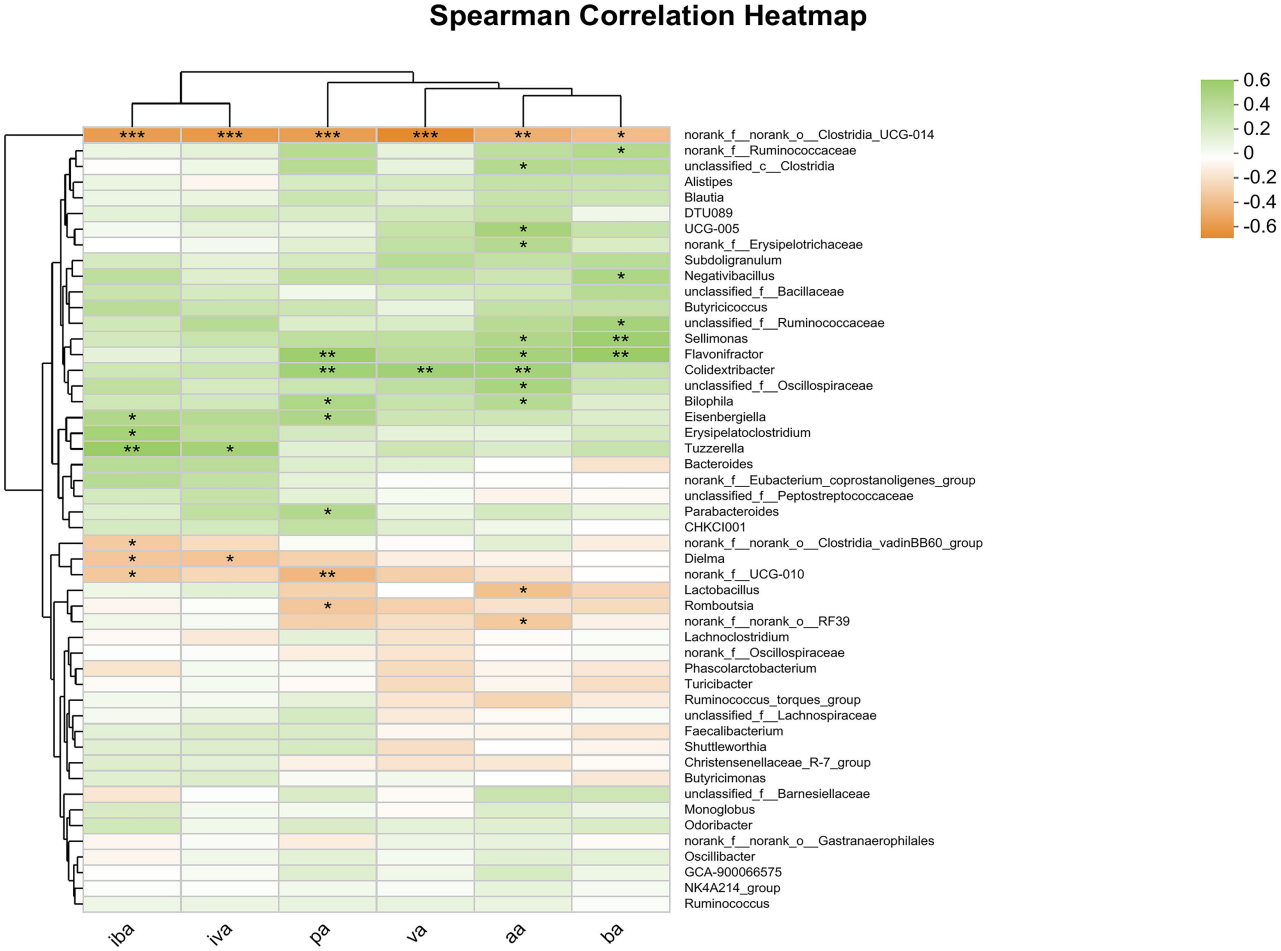
Heatmap of correlation analysis between cecal microorganisms and SCFAs. Statistical differences were recorded as P<0.05 (*), P<0.01 (**) and P<0.001 (***), respectively.

Spearman correlation analysis was conducted at the genus level and a heat map was generated for the cecal microbes and SCFAs in the cecum, including aa, pa, iba, ba, iva, va (**Figure 4**). The abundance of *norank_f_norank_o_Clostridia_UCG-014* was significantly and negatively correlated with the contents of all SCFAs (P<0.05). Additionally, the abundance of *Sellimonas* was significantly and positively correlated with the contents of aa and ba (P<0.05), while the abundance of *Flavonifactor* and *Colidextribacter* was significantly and positively correlated with the contents of aa, pa and ba (P<0.05). The study found significant positive correlation between the abundance of *Parabacteroides* and the content of pa (P <0.05), and significant negative correlation between the abundance of *norank_f_norank_o_Clostridia_vadini BB60_group* and the content of iva (P<0.05). Additionally, other microorganisms were found to be significantly correlated with SCFAs content at the community level.

## 4 Discussion

Although the use of APGs has significant beneficial effects on growth performance and disease prevention in farmed animals, their use is partially restricted and prohibited worldwide due to the increasing problem of bacterial resistance. Studies have reported the beneficial effects of plant polyphenols, flavonoids and polysaccharides on livestock and poultry breeding have been studied and reported (46–48). The results showed that TMAE supplementation could increase the daily gain, improve the serum chemical index, enhance the antioxidant capacity of serum, liver and ileum, enhance the immune function of liver and intestine, and then improve the growth performance of broilers. It indicated that the growth promotion effect of TMAE on broilers was related to a wide range of biological effects. We found that TMAE supplementation had a negative effect on broiler weight gain compared to Con at 1 - 21 days of age. We hypothesized that certain components of TMAE inhibit digestive enzyme activity, which can lead to reduced nutrient uptake in broilers. Digestive enzymes play a crucial role in mediating and limiting energy and nutrient uptake in birds (49). Research has demonstrated that phenolic compounds, including kaempferol and luteolin derivatives, found in dandelion can hinder pancreatic lipase activity (50). Furthermore, dandelion polysaccharides exhibit significant activity in inhibiting α-amylase and α-glucosidase (51). Broiler gut digestion is not optimal in the early stages of growth. Bacteroides, which are responsible for digesting complex carbohydrates in the caecum, begin to proliferate at 14 days (52). During prophase, broilers experienced weight gain inhibition due to their inability to fully digest and utilize polysaccharides in TMAE. The study found that dietary supplementation with TMAE led to an increase in daily weight gain during the 22 - 42 day period. This is a critical stage where productivity increases significantly, resulting in a terminal body weight that exceeds the Con group. The limited energy of TMAE may be the reason for its effect on broilers in the early stages of production. However, the cecal flora in the later stages is capable of fermenting polysaccharides into low molecular weight organic matter that can be utilized. As a result, broilers overcome the energy limitation and exhibit compensatory growth response (53). Broilers consume excessive amounts of energy to maintain compensatory growth. This explains their increased feed intake. In addition, TMAE contains soluble carbohydrates, L-malic acid and 1,3-Dihydroxyacetone dimer, etc., which have obvious sweet smell, improve feed palatability and stimulate appetite of broilers (30, 54–56). The results of serum biochemistry showed that the ALP content of High group was significantly increased (P<0.05). During bone growth and development, bone cells became active and produced a large number of ALP into the blood, promoting bone growth and development (57, 58). At this dose, TMAE also reduced CRE in broiler serum (P<0.05) and increased BUN at Medium dose, but the study showed that except UA, other biochemical indicators reflecting renal function lacked specificity in the assessment of renal health in birds, which was closely related to the special physiological system of birds (59). Medium treatment also significantly increased the level of HDL (P<0.05), and enhanced the conversion of cholesterol to bile acids in liver, which increased the oxidative utilization of fatty acids in muscle tissue and improved growth performance (60, 61). The level of TG in serum can reflect the ability of lipid metabolism of broilers. The study found that Low dose TMAE can significantly reduce the level of TG in serum of broilers. TM polysaccharide may contribute to improving the ability of lipid metabolism of broilers (62).

Long-term intake of high-energy-density feed during breeding will cause peroxidation in livestock and poultry (blood and tissues), and then affect the occurrence and process of inflammation (63–65). Exogenous antioxidant supplementation in the right amount can enhance the capacity of the antioxidant system *in vivo*, on the contrary, excessive antioxidants can also disrupt homeostasis (66, 67). TMAE has been identified in a large number of polyphenols (including flavonoids) and polysaccharides (68), compared with other extracts with stronger antioxidant capacity and extraction solvent is more economical and environmentally friendly (36). The polyphenols of TM have also been shown to have good anti-inflammatory activity (69). The antioxidant activity of TMAE *in vitro* was also confirmed in this study. *In vivo* test results showed that TMAE could significantly increase serum T-AOC and CAT content, and significantly reduce MDA content in broilers. Medium and/or High dose could significantly increase the activities of CAT and GSH-Px in liver and ileum. The antioxidant potential of TM is also partially supported by the studies of Zhao et al. and Du et al. (70, 71)., whose results show that TM polysaccharide and flavonoid extracts can improve the defense capacity of antioxidant system *in vivo*, including the effects on CAT, SOD, T-AOC, etc. Due to the absorption properties of polyphenols (flavonoids) and polysaccharides, we focused on the effects of TMAE on the circulatory, hepatic and intestinal innate immune systems. IL-10 has extensive inflammatory and immunosuppressive properties and plays an important role in maintaining innate immunity and inflammatory homeostasis *in vivo* (72, 73). The intestine is the largest immune organ in chicken, and the immune lymphocytes are the densest in the ileum. At the same time, studies have shown that bile acids play an important role in mucosal immune regulation, and bile acids are mainly absorbed in the ileum (74, 75), so this study focused on the mucosal innate immunity of the ileum. Intestinal epithelial cells and mucosa, as the first physical barrier against external factors, are important components of ileal immunity, while Occludin and ZO-1 are essential to maintain the integrity of intestinal mucosal barrier (76). Meanwhile, intestinal antimicrobial effectors TRF, LZM and DEF β1 are important components of intestinal chemical barrier, which can prevent the infection of pathogenic microorganisms (77–79). sIgA, as the major immunoglobulin in intestinal mucosa, can mediate mucosal immune defense against various endogenous and exogenous pathogens (80). In this study, we found that high dose of TMAE can significantly increase the expression of LZM, Occludin, sIgA, TRF, ZO-1, DEF β1 and other immune indicators in the ileum of broilers, significantly improve the physical and chemical barrier defense effect of ileum mucosa, and enhance the intestinal innate immune function of broilers. Mao et al. also found that TM powder could increase the gene expression level of ZO-1 (35). In addition, the histological observation of intestinal morphology showed that low dose of TMAE could effectively increase the villus height and villus/crypt ratio of duodenum and ileum of broilers, and enhance the absorption capacity of broiler intestine, which confirmed the results of growth performance test of broilers.

Intestinal symbiotic flora can ferment and decompose nutrients for absorption and utilization by the body, and also exist as a biological barrier of intestinal innate immunity, maintaining host immune homeostasis through competition (81, 82). Dietary supplementation with different doses of TMAE had different beneficial effects on broilers, and we evaluated the intervention and regulation of different doses of TMAE on intestinal microflora. The results of alpha diversity analysis based on OUT showed that TMAE supplementation significantly reduced the diversity of broiler intestinal microbiota, but beta diversity analysis and microbiota composition analysis showed that TMAE did not change the composition of intestinal microbiota. We also found that *Lactobacillus aviarius* was gradually enriched with the increase of TMAE addition. *Lactobacillus aviarius* is one of the most common lactobacilli in poultry intestine (83), which can improve intestinal immunity (84). The glucanotransferase secreted by *Lactobacillus aviarius* also has the ability to digest and decompose starch to produce linear α-glucan products (soluble dietary fiber), and can also improve feed utilization rate and promote growth (85, 86). The increase of relative abundance of *Lactobacillus aviarius* may be one of the important reasons why TMAE affects the immune and growth performance of ileum TMAE could increase the relative abundance of Bacteroidetes in caecum. Studies have shown that Bacteroidetes has the ability to rapidly degrade polysaccharides, and can encode a variety of enzymes, such as polysaccharide lyases and glycosidases, to hydrolyze polysaccharides that are difficult to digest to provide energy for the body (87, 88). The results indicated that the polysaccharides in TMAE could affect the composition of intestinal flora in cecum of broilers and increase the relative abundance of Bacteroides. At the genus level, TMAE increased the abundance of *Alistipes* and *Lactobacillus*, and the abundance of *Alistipes* was closely related to body weight, energy metabolism and fat deposition in broilers (89–91). *Alistipes* are involved in the metabolism of SCFAs, and high fat animal diets can increase the relative abundance of the genus, thereby improving lipid metabolism by modulating acetate production (92). In addition, *Alistipes* also had a positive effect on bone development (93), which was consistent with the results of this study that TMAE increased serum alkaline phosphatase content and promoted bone development in broilers. *Alistipes* are also associated with immune regulation and health homeostasis and are considered potentially beneficial bacteria (94). *Lactobacillus* is ubiquitous in the intestinal tract of animals. It can improve the health of animals by inhibiting bacteria, improving the ecological environment of intestinal microorganisms, strengthening the barrier layer of intestinal epithelial cells and improving the immune function of animals (95, 96). Studies have shown that the prebiotic properties of polyphenols and polysaccharides can increase the abundance of probiotics such as *Lactobacillus* and *Bifidobacterium*. *Clostridia*, the most significant differential microbiota produced by TMAE supplementation, not only helps digest food, strengthens the intestinal mucosal barrier, but also provides infection resistance (97, 98). The addition of TMAE could increase the proportion of beneficial bacteria in the cecum of broilers, and reduce the proportion of harmful bacteria such as *Colidotribacter* and *Sellimonas* related to intestinal inflammation (99, 100).

SCFAs are secondary products produced by fermentation of carbohydrates by cecal microorganisms, which can provide necessary energy for host metabolism, improve digestive enzyme activity, inhibit the growth of pathogenic bacteria, and stimulate immune system (101, 102), and play an extremely important role in maintaining intestinal health and function. Aa has the function of regulating intestinal pH and promoting the production of ba, which can promote the regeneration and differentiation of intestinal epithelial cells and improve intestinal morphology (103, 104). The results of this study showed that medium dose of TMAE could significantly increase the content of aa and ba in the cecum of broilers. Correlation heat maps showed that the abundance of *Flavonifractor* was significantly positively correlated with aa and ba content, and it was mainly enriched in the cecal samples of the middle dose group of TMAE. *norank_f:norank_o:Clostridia_UCG-014* was the most abundant in the high dose group and was significantly negatively correlated with all SCFAs, and the content of all SCFAs in the high dose group decreased, which was also consistent with the results. It has been reported that when the host is in immune activation state, intestinal commensal bacteria can change intestinal metabolism, reduce SCFAs and increase the concentration of aromatic metabolites (105). Therefore, it can be speculated that *Clostridia* plays an important role in the activation of ileal innate immunity exhibited after high dose TMAE supplementation. In conclusion, dietary supplementation of TMAE in broilers showed varying degrees of health benefits (improvements in serum biochemistry, antioxidant capacity, immune function, and intestinal morphology) and improved growth performance. These effects may be related to the regulation of intestinal microbiota and SCFAs by TMAE, and it is considered that TMAE may be a potential poultry feed additive capable of replacing APGs. Dietary addition of TMAE at 1000 mg/kg may have a broader beneficial biological effect.

## Conclusions

Supplementing broiler diets with TMAE at varying doses was found to enhance growth performance and overall health. The most significant benefits were observed at a dose of 1000 mg/kg, including improved serum biochemical parameters, intestinal morphology, antioxidant capacity of the liver and ileum, immune function of the liver and ileum, and increased SCFAs content. *Lactobacillus aviarius*, *norank_f_norank_o:Clostridia_UCG-014*, and *Flavonifractor* may have a dominant role in the microflora of the intestine and cecum.

## Data availability statement

The raw data supporting the conclusions of this article will be made available by the authors, without undue reservation. Accession number of NIH BioProject: PRJNA1052070.

## Ethics statement

The animal study was approved by Animal Ethics Committee of Hunan Agricultural University. The study was conducted in accordance with the local legislation and institutional requirements.

## Author contributions

ZD: Conceptualization, Data curation, Formal analysis, Methodology, Software, Validation, Visualization, Writing – original draft, Writing – review & editing. ZL: Methodology, Writing – review & editing. YX: Methodology, Writing – review & editing. BT: Data curation, Formal analysis, Methodology, Software, Visualization, Writing – original draft. WS: Methodology, Writing – review & editing. QA: Investigation, Resources, Supervision, Writing – review & editing. ZY: Investigation, Methodology, Project administration, Supervision, Writing – review & editing. JZ: Conceptualization, Funding acquisition, Project administration, Resources, Supervision, Writing – review & editing.
