# Supplementary material for: Potential for the development of Taraxacum mongolicum aqueous extract as a phytogenic feed additive for poultry
Source: Front Immunol. 2024 Mar 11;15:1354040. doi: 10.3389/fimmu.2024.1354040 (PMC10961442; doi:10.3389/fimmu.2024.1354040)
Supplement: Supplementary file 1 [file DataSheet_1.docx]

Supplementary Material


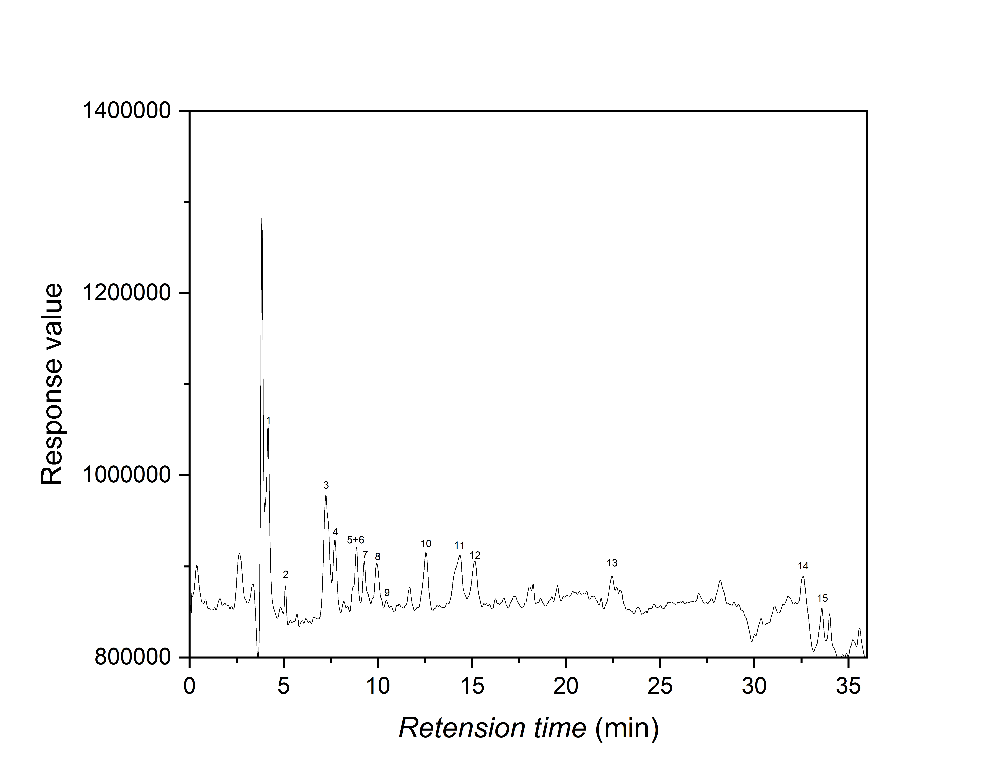


**Supplementary Figure 1.** Identification of TMAE.

**Table S1** Main Phenolic Compounds in TMAE

| **Peak** | **RT** | **m/z** | **Compound names** |
| --- | --- | --- | --- |
| 1 | 4.09 | 179.055 | 1,3-Dihydroxyacetone dimer |
| 2 | 4.21 | 133.014 | L-Malic acid |
| 3 | 7.09 | 256.966 | 5,7-Dihydroxyflavanone |
| 4 | 7.44 | 311.042 | Caftaric acid |
| 5 | 8.65 | 261.078 | violaceol-II |
| 6 | 8.84 | 253.073 | Chrysophanol |
| 7 | 9.24 | 137.024 | Protocatechuic aldehyde |
| 8 | 9.89 | 177.020 | Esculetin |
| 9 | 10.13 | 179.035 | Caffeic acid |
| 10 | 12.39 | 593.156 | Luteolin 7-rutinoside |
| 11 | 14.42 | 473.075 | Chicoric acid |
| 12 | 15.03 | 447.097 | Luteolin 7-O-glucoside |
| 13 | 22.47 | 740.499 | Unknown |
| 14 | 32.69 | 285.042 | Luteolin |
| 15 | 33.42 | 167.0354 | vanillic acid |
